# Protective Effects of Natural Products, Functional Foods, and Probiotics on NSAID-Induced Small Intestinal Injury: A Systematic Review with Mechanistic Considerations of Oxidative Stress and Microbiome Modulation

**DOI:** 10.3390/antiox15070903

**Published:** 2026-07-21

**Authors:** Ji Hye Hwang, You-Kyung Choi

**Affiliations:** 1 Department of Acupuncture & Moxibustion Medicine, College of Korean Medicine, Gachon University, Seongnam 13120, Republic of Korea; jhbori@nate.com; 2 Department of Korean Internal Medicine, College of Korean Medicine, Wonkwang University, Iksan 54538, Republic of Korea

**Keywords:** NSAID, aspirin, small intestinal injury, enteropathy, natural products, probiotics, capsule endoscopy, intestinal permeability, systematic review

## Abstract

NSAID-induced small intestinal injury is a clinically significant complication among chronic NSAID and aspirin users, yet effective treatment options remain limited. This systematic review evaluated natural products, functional foods, and probiotics for preventing or treating NSAID-induced small intestinal injury. PubMed, Embase, CENTRAL, and CNKI were searched from inception to January 2026 for randomized studies involving adults with NSAID- or aspirin-induced enteropathy assessed using capsule endoscopy or intestinal permeability testing. Risk of bias was assessed using RoB 2 and ROBINS-I. Due to substantial clinical and methodological heterogeneity, meta-analysis was not performed, and findings were synthesized narratively. Twenty-two studies were included: 21 randomized controlled trials and one quasi-randomized study. Geranylgeranylacetone demonstrated protective effects in three of four capsule endoscopy studies. Lactoferrin, zinc carnosine, and fish protein hydrolysate reduced indomethacin-induced intestinal hyperpermeability. Probiotic effects appeared outcome-dependent, with more consistent benefits observed for capsule endoscopy-based mucosal injury outcomes than for intestinal permeability outcomes. Among randomized trials, three were rated as having low risk of bias, 15 had some concerns, and three had high risk. Overall, preliminary evidence suggests that selected natural-origin interventions may protect against NSAID/aspirin-induced small intestinal injury. However, because the certainty of evidence was generally low or very low, these findings should be interpreted as hypothesis-generating and require confirmation in larger, methodologically rigorous trials.

## 1. Introduction

Nonsteroidal anti-inflammatory drugs (NSAIDs) and aspirin are among the most widely prescribed medications worldwide, with indications ranging from analgesia and anti-inflammatory therapy to cardiovascular prophylaxis [1,2,3]. Despite their well-established clinical utility, chronic NSAID and aspirin use is associated with substantial gastrointestinal toxicity [1,2,3,4]. Although gastroduodenal complications have been extensively studied and are partially mitigated by proton pump inhibitors (PPIs), the small intestine has emerged as a distinct and underrecognized site of NSAID-induced injury [2,3,5]. With the widespread adoption of video capsule endoscopy (VCE) and magnetically controlled capsule endoscopy (MCCE) over the past two decades, it has become increasingly apparent that small intestinal mucosal injury—including erosions, ulcers, and mucosal breaks—occurs in over 50% of chronic NSAID and aspirin users, with clinically significant complications including occult bleeding, iron deficiency anemia, and protein-losing enteropathy reported in a substantial proportion of affected patients [2,3,4,6,7].

The pathogenesis of NSAID-induced small intestinal injury is multifactorial and differs from that of gastroduodenal injury [2]. The primary mechanisms involve uncoupling of mitochondrial oxidative phosphorylation within enterocytes, resulting in intracellular adenosine triphosphate (ATP) depletion and generation of reactive oxygen species (ROS). These processes jointly disrupt tight junction integrity, increase paracellular permeability, and expose the mucosa to luminal aggressive factors, including bacteria, bile acids, and food-derived antigens [4,8,9,10,11]. Unlike gastroduodenal injury, which is predominantly mediated by cyclooxygenase-1 inhibition and the consequent reduction in gastroprotective prostaglandins, small intestinal injury involves both topical and systemic mechanisms and is not prevented by PPIs [2,12]. Moreover, emerging evidence suggests that PPI coadministration may paradoxically exacerbate small intestinal injury by altering the gut microbiome and increasing luminal bacterial load, further highlighting the need for alternative preventive and therapeutic strategies specifically targeting the small intestine [3,5,13].

Current management options for NSAID-induced small intestinal injury remain limited [2,3]. Misoprostol, a synthetic prostaglandin analog, has demonstrated efficacy in reducing small intestinal mucosal lesions but is associated with significant gastrointestinal adverse effects that limit its clinical acceptability [14,15]. Rifaximin, a poorly absorbed antibiotic, has shown potential for reducing the severity of NSAID-induced small intestinal lesions, possibly through modulation of the gut microbiome [3,16]. Rebamipide, a mucosal protective agent, has been investigated primarily in Asian populations [3,17]. A recent systematic review by Choe et al. (2024) comprehensively evaluated synthetic pharmaceutical interventions for NSAID-induced small intestinal injury and highlighted the overall paucity of high-quality evidence supporting any single therapeutic agent [3].

Against this background, there has been growing interest in the potential role of natural products, functional foods, and probiotic microorganisms in the prevention or treatment of NSAID-induced small intestinal injury [2,3]. These interventions offer several theoretical advantages, including favorable safety profiles, mechanistic plausibility through antioxidant, anti-inflammatory, barrier-protective, and microbiome-modulatory pathways, and alignment with patient preferences for natural therapeutic approaches [2]. Several individual studies have investigated a diverse range of such interventions, including herbal compounds such as geranylgeranylacetone (GGA) and polaprezinc [18,19]; protein-derived functional foods such as lactoferrin and fish protein hydrolysate [20,21]; nutraceuticals such as zinc carnosine and glutamine [22,23]; and probiotic strains of *Lactobacillus* and *Bifidobacterium* [24,25]. However, to date, no systematic review has comprehensively synthesized the evidence across this diverse group of interventions. Aspirin was included alongside NSAIDs because low-dose aspirin shares overlapping pathogenic pathways with conventional NSAID enteropathy and has been specifically investigated in several pivotal trials in this field [3,4]. The broad intervention scope reflects an intentional evidence-mapping approach, designed to identify preliminary signals across a clinically underexplored area rather than to generate definitive efficacy conclusions for any single intervention class.

The present systematic review aimed to comprehensively identify, critically appraise, and synthesize evidence from randomized controlled trials evaluating the protective effects of natural products, functional foods, and probiotics on NSAID-induced small intestinal injury.

## 2. Materials and Methods

### 2.1. Protocol and Registration

This systematic review was conducted and reported in accordance with the Preferred Reporting Items for Systematic Reviews and Meta-Analyses (PRISMA) 2020 guidelines [26]. The review protocol was prospectively registered with the International Prospective Register of Systematic Reviews (PROSPERO; registration no. CRD420261355613). Methodological refinements made during the review process, including the decision to synthesize the evidence narratively rather than quantitatively, are described in the relevant Methods sections below.

### 2.2. Eligibility Criteria

#### 2.2.1. Study Design

RCTs were eligible for inclusion in the primary synthesis. One quasi-randomized crossover study [23], identified during full-text review, was retained for narrative synthesis as supportive mechanistic evidence and was assessed separately using Risk of Bias in Non-randomized Studies of Interventions (ROBINS-I) [27], as described in Section 2.8. Nonrandomized studies, observational studies, case reports, review articles, editorials, animal studies, and conference abstracts without retrievable data were excluded. Conference abstracts containing sufficient extractable data were included when the corresponding full-text article was unavailable, with this limitation transparently reported.

#### 2.2.2. Participants

Studies involving adults (aged ≥18 years) receiving NSAIDs or aspirin either for clinical indications or as part of experimentally induced NSAID/aspirin injury models in healthy volunteer studies were eligible. Studies involving patients with pre-existing inflammatory bowel disease, celiac disease, or other conditions primarily affecting small intestinal integrity were excluded to ensure that the observed effects were attributable to NSAID-induced injury rather than to the underlying disease.

#### 2.2.3. Interventions

Studies evaluating any natural product, functional food, or probiotic/synbiotic/prebiotic intervention were eligible. This review adopted a broad natural-origin framework, defining eligible interventions as compounds derived from, or structurally based on, naturally occurring substances. These included plant-derived compounds, animal-derived functional foods (such as lactoferrin and fish protein hydrolysate), naturally derived mineral complexes, amino acid derivatives, nucleotides, dietary fibers, and probiotic microorganisms.

Accordingly, GGA (a terpenoid compound structurally derived from natural isoprenoids) and polaprezinc (a chelate of zinc and the naturally occurring amino acid derivative L-carnosine) were classified as natural-origin interventions and included in Group A. We acknowledge that these agents may also be viewed as pharmaceutical or semi-synthetic mucosal protective agents in some clinical contexts; therefore, their classification within the broad natural-origin/complementary intervention framework is explicitly stated to avoid confusion. Studies evaluating synthetic pharmaceuticals only (including rebamipide, misoprostol, PPIs, rifaximin, and nonsteroidal comparators) as the primary intervention were excluded.

#### 2.2.4. Comparators

Eligible comparators included placebo, no treatment, or active control with a pharmacological agent, provided that the natural product or probiotic intervention constituted the primary comparison of interest.

#### 2.2.5. Outcomes

The primary outcomes were: (1) endoscopic assessment of small intestinal mucosal injury using VCE or MCCE, including mucosal break count, Lewis score, and capsule endoscopy score; and (2) intestinal permeability assessed using urinary sugar excretion tests, primarily the lactulose-to-mannitol (L/M) ratio or lactulose-to-rhamnose (L/R) ratio. For simplicity, VCE and MCCE findings are collectively referred to as capsule endoscopy-based outcomes throughout this manuscript. Studies reporting only subjective symptom scores without objective assessment of mucosal injury or intestinal permeability were excluded.

### 2.3. Search Strategy

A systematic electronic search was conducted in PubMed (including MEDLINE), Embase, the Cochrane Central Register of Controlled Trials (CENTRAL), and the CNKI from database inception to January 2026, without language restrictions. Supplementary Table S1 provides the full search strategies for each database. Briefly, the search combined terms related to NSAIDs and aspirin with terms related to small intestinal injury and relevant outcome measures (capsule endoscopy, intestinal permeability, erosion, ulcer, and mucosal break). For CENTRAL, which indexes only RCTs, no additional randomization filter was applied. For CNKI, searches were conducted using Chinese-language subject terms and keywords. Conference abstract databases were searched through Embase. Clinical trial registries (ClinicalTrials.gov, the World Health Organization [WHO] International Clinical Trials Registry Platform [ICTRP], and the Clinical Research Information Service of Korea) were also searched to identify ongoing or unpublished trials. In addition, the reference lists of included studies and relevant systematic reviews were manually screened to identify further eligible studies. Korean-language databases (KISS, OASIS, and KMbase) were additionally searched. Records related to NSAID/aspirin-associated small intestinal injury were retrieved; however, no eligible studies were identified.

### 2.4. Study Screening and Selection Process

All search results were imported into EndNote™ 20 reference management software, and duplicate records were removed before screening. Title and abstract screening were performed independently by two reviewers according to pre-specified eligibility criteria. Discrepancies were resolved by consensus or consultation with a third reviewer. Full-text articles were retrieved for all records that passed the title/abstract screening, and eligibility was confirmed through independent full-text review. A PRISMA 2020 flow diagram [26] was constructed to document the study selection process.

### 2.5. Data Extraction

Data were extracted independently by two reviewers using a standardized data extraction form. Extracted variables included study design and setting; participant characteristics (sample size, age, sex, indication for NSAID/aspirin use); intervention details (compound, dose, formulation, route of administration, and duration); comparator(s); NSAID/aspirin type and dose; primary and secondary outcomes; and key findings. For crossover trials, we extracted information on the washout period, whether carryover effects were explicitly assessed by the original authors, and whether the reported results were based on first-period, post-crossover, or combined crossover data. Where first-period data were available, these were preferentially considered to minimize potential carryover effects. When only post-crossover or combined data were reported, this was transparently noted in the study characteristics table and considered when interpreting the findings. Corresponding authors of studies available only as conference abstracts were contacted via email to request additional information. In cases of duplicate publications derived from the same dataset, the most complete and/or peer-reviewed publication was retained.

### 2.6. Risk of Bias Assessment

The risk of bias for all included RCTs was assessed independently by two reviewers using the Cochrane Risk of Bias 2 (RoB 2) tool [28]. This tool evaluates five domains: (D1) bias arising from the randomization process; (D2) bias due to deviations from intended interventions (including blinding of participants and personnel); (D3) bias due to missing outcome data; (D4) bias in outcome measurement (including blinding of outcome assessors); and (D5) bias in selection of the reported result. Overall risk of bias was rated as low, some concerns, or high. For the quasi-randomized study [23], the ROBINS-I tool [27] was applied instead, as it did not meet criteria for random allocation. Any discrepancies between reviewers were resolved through discussion and consensus.

### 2.7. Data Synthesis

Given the substantial clinical and methodological heterogeneity across interventions, NSAID/aspirin exposure models, study populations, outcome domains, and study designs, a quantitative meta-analysis was not conducted. To illustrate the extent of clinical heterogeneity, the included studies differed in multiple dimensions, including intervention origin and proposed mechanism (e.g., natural-origin mucosal protective agents, mineral or amino acid-related compounds, functional food-derived proteins, dietary fibres, and probiotic microorganisms), NSAID/aspirin agent (indomethacin, aspirin, diclofenac, or celecoxib), injury model (short-term experimental injury models in healthy volunteers versus treatment of established injury in chronic users), primary outcome domain (capsule endoscopy-based mucosal injury versus intestinal permeability), outcome scale (mucosal break count, Lewis score, capsule endoscopy score, L/M ratio, or L/R ratio), study duration (5 days to 12 weeks), and data availability (several crossover trials reported only combined data without paired within-period estimates). This multidimensional heterogeneity precluded meaningful quantitative synthesis even within nominally similar subgroups. Therefore, this review was designed to map and synthesize the available evidence by intervention category and outcome domain, rather than to generate a single pooled effect estimate. A narrative synthesis was conducted in accordance with the Synthesis Without Meta-analysis (SWiM) reporting guidelines [29]. Studies were first categorized into two primary groups based on intervention type: Group A (natural products and functional foods) and Group B (probiotics and synbiotics). Within each group, studies were further organized by outcome domain, including capsule endoscopy-based mucosal injury outcomes and intestinal permeability outcomes, to facilitate structured comparison. The synthesis did not rely solely on statistical significance. Direction of effect, reported magnitude of effect where available, clinical context, study design, risk of bias, and consistency across studies were considered. To further contextualize the findings, we conducted a GRADE-informed certainty assessment by intervention category and outcome domain. Certainty ratings considered risk of bias, inconsistency, indirectness, imprecision, and publication bias. Because quantitative pooling was not performed, certainty ratings were based on a narrative assessment of the overall body of evidence rather than pooled effect estimates.

### 2.8. Deviations from Registered Protocol

Several minor deviations from the registered protocol are reported for transparency. First, although meta-analysis was initially planned, the substantial clinical and methodological heterogeneity across included studies—including diversity of interventions, NSAID agents, populations, and outcome measures—precluded meaningful quantitative synthesis; a narrative synthesis was therefore conducted in accordance with the SWiM reporting guidelines [29]. Second, one non-randomized crossover study [23] identified during full-text review was assessed using ROBINS-I [27] rather than RoB 2 [28], as it did not meet the criteria for randomized allocation. This study was retained for narrative synthesis as supportive mechanistic evidence only and excluded from any primary comparative analyses. Third, one eligible study, available only as a conference abstract, was included, given the availability of sufficient data for extraction, with this limitation transparently reported. Fourth, clinical trial registries (ClinicalTrials.gov, WHO ICTRP, and the Korean Clinical Research Information Service) were searched in addition to the pre-specified electronic databases to identify unpublished or ongoing trials. Four registered trials without published results were identified and reported as excluded studies. These deviations did not substantially alter the scope or conclusions of the review.

## 3. Results

### 3.1. Study Selection

The electronic database search identified 438 records: PubMed (*n* *=* 96), Embase (*n =* 149), CENTRAL (*n =* 153), and CNKI (*n =* 40). After removing 224 duplicate records, 214 records underwent title and abstract screening. Of these, 178 records were excluded based on predefined eligibility criteria, primarily because they evaluated synthetic pharmaceutical interventions only, employed animal study design, were systematic or narrative reviews, assessed gastroduodenal rather than small intestinal endpoints, or did not include a therapeutic or preventive intervention.

**Figure 1:**
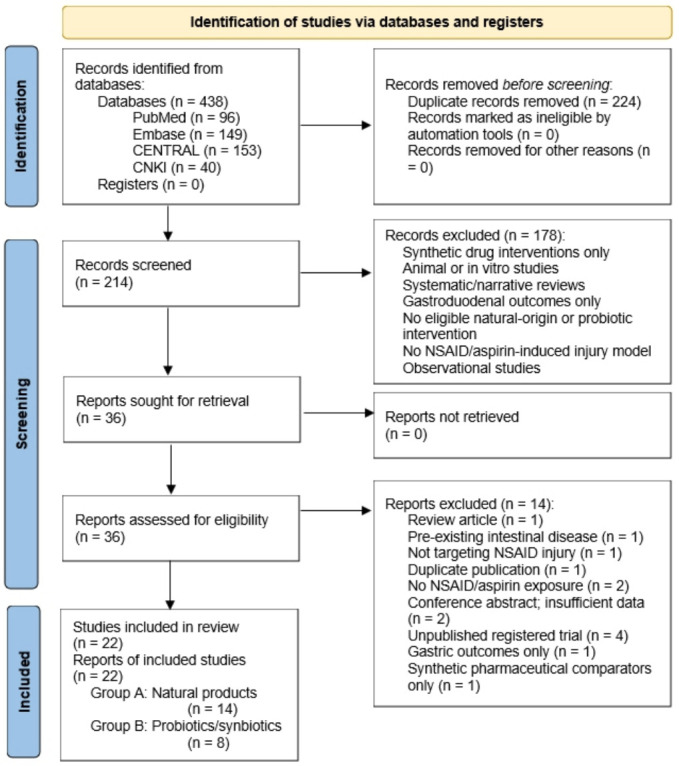
The Preferred Reporting Items for Systematic Reviews and Meta-Analyses 2020 Flow Diagram.

Full-text review was performed for 36 records, of which 14 were subsequently excluded for the following reasons: one review article; one study involving patients with Crohn’s disease rather than NSAID-induced injury; one study in which the cranberry beverage intervention was not specifically directed at NSAID-induced injury; one duplicate publication of an already included dataset; two studies in which no NSAID was used as the injurious agent; two studies available as conference abstracts only with insufficient data for inclusion despite author contact; four registered trials with no published results available at the time of the search (NCT05993247, KCT0006208, KCT0000080, NCT04447924); one study assessing gastric rather than small intestinal outcomes; and one study assessing only synthetic pharmaceutical comparators. Ultimately, 22 studies [18,19,20,21,22,23,24,25,30,31,32,33,34,35,36,37,38,39,40,41,42,43] were included in the final synthesis. The study selection process is summarized in the PRISMA flow diagram (Figure 1).

This flow diagram illustrates the systematic search and study selection process. A total of 438 records were initially identified, of which 224 duplicates were removed. Following title and abstract screening of the remaining 214 records, 36 full-text articles were assessed for eligibility. Ultimately, 22 studies met the inclusion criteria, including 21 randomized controlled trials and one quasi-randomized study. In the screening and eligibility stages, “wrong organism” refers to non-human studies, including animal or in vitro studies, and “wrong comparator” refers to studies in which the comparator or comparison structure did not meet the eligibility criteria for this review.

### 3.2. Overview of Study Characteristics

The 22 included studies [18,19,20,21,22,23,24,25,30,31,32,33,34,35,36,37,38,39,40,41,42,43] were conducted across eight countries: Japan (*n =* 9), the Netherlands (*n =* 5), China (*n =* 3), the United Kingdom (*n =* 2), Denmark, Belgium, Sweden, and the United States (*n =* 1 each). The studies were published between 1999 and 2024. The NSAIDs used as injury-inducing agents included indomethacin (*n =* 9) [20,21,22,23,35,36,37,41,42], aspirin (*n =* 8) [19,24,25,30,34,38,40,43], diclofenac (*n =* 4) [18,31,32,33], and celecoxib (*n =* 1) [39]. Sample sizes ranged from 10 to 60 participants. Fourteen studies employed a crossover design, while eight used a parallel-group design. Table 1 presents detailed characteristics of included studies.

The studies were categorized into two groups: Group A, comprising 14 studies evaluating natural products and functional foods [18,19,20,21,22,23,30,31,32,33,34,35,36,37], and Group B, comprising eight studies evaluating probiotics and synbiotics [24,25,38,39,40,41,42,43]. Within each group, studies were further stratified according to outcome measure. In Group A, seven studies used VCE as the primary outcome (VCE subgroup) [18,19,30,31,32,33,34], and seven used intestinal permeability testing (permeability subgroup) [20,21,22,23,35,36,37]. In Group B, five studies used VCE [24,25,38,39,40] and three used permeability testing [41,42,43]. Furthermore, the included studies encompassed two clinically distinct scenarios (Table 1): short-term prevention models in healthy volunteers with experimentally induced NSAID/aspirin injury (*n =* 17), and treatment-oriented models in chronic aspirin/NSAID users with pre-existing mucosal injury (*n =* 5).

### 3.3. Risk of Bias

**Figure 2:**
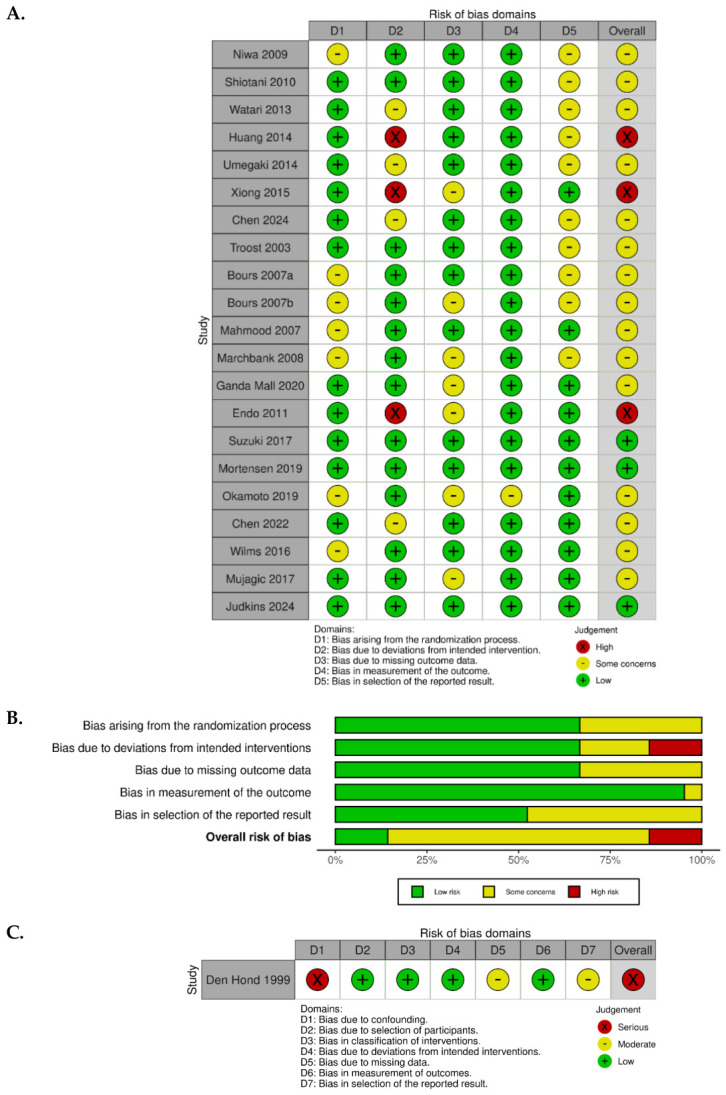
Risk of Bias Assessment. (**A**) Risk-of-bias summary (traffic light plot) showing domain-level judgments for the 21 randomized controlled trials assessed using the Cochrane Risk of Bias 2 tool [28]. (**B**) Weighted bar chart displaying the proportion of risk-of-bias judgments across all randomized trials. (**C**) Risk-of-bias assessment for the quasi-randomized study using the ROBINS-I tool [27]. The studies evaluated in this figure correspond to the following references: Niwa 2009 [18], Shiotani 2010 [30], Watari 2013 [19], Huang 2014 [31], Umegaki 2014 [32], Xiong 2015 [33], Chen 2024 [34], Troost 2003 [20], Bours 2007a [35], Bours 2007b [36], Mahmood 2007 [22], Marchbank 2008 [21], Ganda Mall 2020 [37], Endo 2011 [24], Suzuki 2017 [38], Mortensen 2019 [25], Okamoto 2019 [39], Chen 2022 [40], Wilms 2016 [41], Mujagic 2017 [42], Judkins 2024 [43], and Den Hond 1999 [23].

Figure 2 summarizes risk of bias assessments. Among the 21 RCTs, three (14%) were rated as having an overall low risk of bias [25,38,43], 15 (71%) as having some concerns, and three (14%) as having a high risk of bias [24,31,33]. One quasi-randomized crossover study [23] was assessed separately using the ROBINS-I tool [27] and rated as having a serious risk of bias.

The three high-risk studies shared a common limitation: the absence of a placebo control and inadequate blinding of participants and personnel, resulting in a high risk of bias due to deviations from intended interventions. Among studies rated as having some concerns, the most frequent issues were incomplete reporting of allocation concealment procedures, absence of prospective trial registration, and, in some cases, differential attrition. In several open-label VCE studies [19,34,40], the overall risk of bias was judged as “some concerns” rather than “high risk” because mucosal injury outcomes were assessed by blinded independent endoscopists, partially mitigating concerns related to outcome measurement.

The quasi-randomized crossover study [23] was assessed using ROBINS-I [27] because the experimental conditions were not performed in random order. It was rated as having a serious risk of bias primarily due to uncontrolled order and period effects, which may have introduced systematic confounding.

### 3.4. Group A: Natural Products and Functional Foods

#### 3.4.1. VCE Subgroup (*n*= 7)

GGA, a terpenoid compound with mucosal cytoprotective and heat shock protein-inducing properties, was the most extensively studied natural product in the VCE subgroup, with four studies reporting its effects. One double-blind RCT [18] conducted in 40 participants receiving diclofenac demonstrated a significant reduction in combined gastric and small intestinal injury scores in the GGA group compared with placebo (*p* = 0.027), although the reduction in small intestinal scores alone did not reach statistical significance. In another RCT [32] of 42 diclofenac-treated participants, GGA was compared with famotidine and was associated with significantly fewer erosions (*p* = 0.032) and ulcers (*p* = 0.017). A prospective randomized study [33] involving diclofenac-treated 60 participants also reported significantly lower post-treatment Lewis scores in the GGA group compared with controls (*p* = 0.017). In contrast, one pilot study [30] did not find a significant effect of GGA in aspirin-treated participants; the authors attributed this finding to the use of a lower-dose aspirin model and potential differences in NSAID-induced injury mechanisms.

Polaprezinc (a zinc-carnosine complex) was evaluated in an open-label RCT [19] involving 36 aspirin users and demonstrated a significant within-group reduction in erosion scores (*p* = 0.039); however, no between-group comparison was reported, limiting the interpretability of this finding. Muscovite clay (3 g twice daily) significantly reduced the incidence of mucosal breaks in diclofenac-treated participants compared with controls (31.3% vs. 71.4%; *p* = 0.028) in one investigation [31]; however, the open-label design and absence of a placebo control introduced potential bias. A recent trial [34] evaluating a combination preparation containing glutamine and herbal components in aspirin users reported significant improvements in endoscopic injury scores (*p* < 0.001) and higher mucosal healing rates (78.9% vs. 5.6%) compared with controls, although this study was also limited by the absence of blinding.

#### 3.4.2. Permeability Subgroup (*n* = 7)

Lactoferrin (5 g/day for 7 days), a multifunctional iron-binding glycoprotein with anti-inflammatory and mucosal barrier-protective properties, significantly attenuated indomethacin-induced increases in the intestinal permeability ratio compared with placebo in a double-blind crossover RCT [20]. This was assessed using the L/R ratio, which was lower in the lactoferrin group (0.036 vs. 0.028; *p* < 0.05). This study was rated as having some concerns overall, primarily due to the absence of prospective trial registration, while other domains were assessed as low risk.

Zinc carnosine (37.5 mg twice daily) prevented the indomethacin-induced increase in intestinal permeability in a crossover RCT [22]. In the placebo arm, the L/M ratio increased significantly (0.35 to 0.88; *p* < 0.01), while zinc carnosine co-administration prevented this increase. Similarly, fish protein hydrolysate (1 g three times daily) significantly attenuated indomethacin-induced permeability increases compared with placebo (L/M ratio: 0.35 to 0.59 vs. 0.28 to 1.54; *p* < 0.01) in another crossover trial [21].

ATP administered directly into the small intestine via naso-jejunal infusion significantly reduced indomethacin-induced permeability (L/R ratio: 0.042 to 0.027; *p* < 0.01) in one investigation [36]. However, a parallel study by the same group found no significant effect with enteric-coated oral ATP capsules [35]; the authors attributed this difference to the mismatch between oral dosing and the site of active ATP release. Glutamine supplementation in one study [23] demonstrated a significant preventive effect only when administered concurrently with indomethacin, rather than prior to exposure (*p* = 0.001); however, this study was assessed using ROBINS-I due to its quasi-randomized design and was rated as having a serious risk of bias. Finally, a nutritional study investigating dietary fiber supplementation (12 g/day) [37] did not significantly reduce indomethacin-induced permeability in an elderly population, with the authors suggesting that reduced colonic fermentation capacity in older individuals may have limited its efficacy.

### 3.5. Group B: Probiotics and Synbiotics

#### 3.5.1. VCE Subgroup (*n* = 5)

All five probiotic studies that employed VCE as the primary outcome measure demonstrated statistically significant reductions in mucosal injury. One investigation [24] reported significant reductions in capsule endoscopy scores following *Lactobacillus casei* Shirota supplementation in aspirin users (*p* = 0.026). A double-blind, placebo-controlled crossover RCT [38] demonstrated a significant reduction in mucosal breaks following *L. gasseri* yogurt consumption (*p* < 0.01). Another trial [25], rated as having a low risk of bias, reported a significant reduction in the Lewis score area under the curve (AUC) in high-dose aspirin users treated with *Bifidobacterium breve* Bif195 compared with placebo (3040 vs. 4351; *p* = 0.038). In one study [39], a significantly lower incidence of celecoxib-induced ulcers was found in participants receiving *L. salivarius* WB21 compared with controls (14.8% vs. 40%; *p* = 0.043). Finally, a prospective clinical trial [40] reported significant improvements in endoscopic injury scores following *Lactobacillus* complex supplementation in aspirin users (*p* < 0.001).

#### 3.5.2. Permeability Subgroup (*n* = 3)

In contrast to the consistently positive VCE findings, none of the three probiotic studies using intestinal permeability as the primary outcome demonstrated statistically significant effects. One trial [41] found no significant reduction in indomethacin-induced permeability following synbiotic (*Ecologic 825* + FOS) supplementation, with a notable sex imbalance between groups (*p* = 0.025), which was acknowledged as a study limitation. Another study [42] reported no significant effect of *L. plantarum* supplementation on the L/M ratio, although gene expression analyses suggested partial modulation of tight junction-related pathways. A recent double-blind trial [43] also found no significant effect of combined *L. helveticus* and *L. rhamnosus* supplementation on aspirin-induced permeability, with the authors noting a potential methodological issue involving contamination of the L-rhamnose tracer with neotame.

### 3.6. Summary of Findings

**Figure 3:**
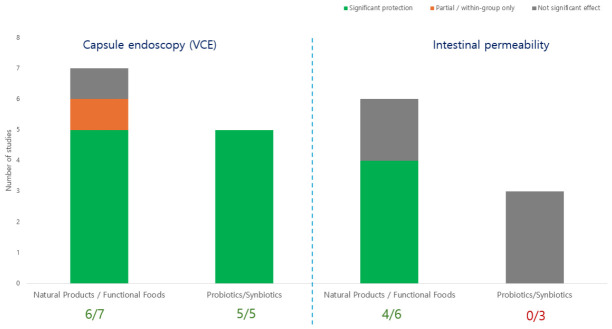
Direction of reported findings stratified by intervention group and outcome domain. Numbers within the bars represent the number of studies reporting favorable findings (green), partial or suggestive findings (orange), or no clear effect (gray). The summary ratios below the bars indicate the number of favorable or partial/suggestive findings relative to the total number of included randomized controlled trials. The study by Den Hond et al. [23] was excluded from the permeability synthesis because of serious risk of bias, as assessed using ROBINS-I. Watari et al. [19] was classified as showing a partial effect because it reported significant within-group improvement without a corresponding between-group comparison. Data were derived from studies evaluating natural products and functional foods [18,19,20,21,22,23,30,31,32,33,34,35,36,37] and probiotics/synbiotics [24,25,38,39,40,41,42,43]. Natural products: VCE, *n =* 7; intestinal permeability, *n =* 6. Probiotics: VCE, *n =* 5; intestinal permeability, *n =* 3. Given the small number of studies in each subgroup, clinical heterogeneity, and varying methodological quality, this observed pattern should be considered hypothesis-generating rather than definitive.

Across both intervention groups, the direction of findings appeared to vary according to outcome domain. In the Group A capsule endoscopy-based subgroup, several studies of natural products and functional foods reported favorable findings [18,19,30,31,32,33,34], although the strength and consistency of evidence varied across interventions and study designs. Specifically, one study reported significance only for a combined gastric and small intestinal endpoint, whereas another reported within-group improvement without a corresponding between-group comparison. In the intestinal permeability subgroup, several randomized trials reported favorable findings [20,21,22,35,36,37], whereas the quasi-randomized glutamine study [23] provided supportive but methodologically limited evidence. Probiotic and synbiotic interventions also appeared to show an outcome-dependent pattern (Figure 3). The available capsule endoscopy-based studies generally reported favorable findings for mucosal injury outcomes [24,25,38,39,40], whereas no clear effect on intestinal permeability outcomes was observed across the three small permeability studies [41,42,43]. This apparent divergence between structural mucosal outcomes and functional permeability outcomes should be interpreted as hypothesis-generating rather than definitive, particularly given the heterogeneity of probiotic strains, study populations, and outcome measures.

Due to substantial heterogeneity in interventions, NSAID/aspirin exposure models, study populations, and outcome domains, a pooled meta-analysis was not performed. Secondary outcomes, including gastrointestinal symptoms and adverse events, were inconsistently reported across the included studies, precluding systematic synthesis. Where reported, no serious adverse events attributable to the study interventions were noted; however, safety remains insufficiently characterized because adverse-event reporting was inconsistent across studies (Table 2).

To further contextualize these narrative findings, a GRADE-informed certainty assessment was conducted by intervention category and outcome domain (Table 3).

To further contextualize these narrative findings, a formal summary of findings table presenting GRADE-informed certainty ratings by intervention category and outcome domain is provided in Table 3. Overall, the certainty of evidence was rated as low or very low across categories, mainly because of risk of bias, clinical heterogeneity, small sample sizes, and imprecision.

### 3.7. Mechanistic Correlates: Oxidative Stress, Inflammation, and Cellular Repair

Although the primary focus of the included studies was macroscopic mucosal injury and functional permeability, several trials provided direct or indirect mechanistic evidence relevant to oxidative stress, intestinal inflammation, and cellular repair pathways. These findings are summarized in Table 4 and described below according to three levels of evidence: direct biomarker measurements, transcriptomic evidence, and the proposed mechanisms cited by the trial authors on the basis of preclinical models.

Only one study [37] directly quantified systemic oxidative stress as a prespecified secondary outcome. This study measured plasma hydrogen peroxide concentrations using the Free Oxygen Radicals Test and demonstrated a significant reduction following 6 weeks of oat beta-glucan supplementation (2.04 to 1.76 mmol/L; *p* = 0.0005), suggesting a systemic antioxidant effect that was dissociated from the intestinal permeability endpoint. Notably, this was the only study in the present review to report a directly measured antioxidant outcome.

Two studies assessed intestinal inflammation using fecal calprotectin. One randomized trial [25] demonstrated that *B. breve* Bif195 supplementation was associated with a significantly lower AUC for fecal calprotectin compared with placebo during aspirin challenge (*p* = 0.0347), indicating reduced mucosal neutrophil infiltration. Another study [32] demonstrated that the GGA group showed a significantly smaller post-treatment calprotectin increase compared with the famotidine control group (between-group *p* = 0.041), with neither group exhibiting a significant within-group increase. This finding provides indirect clinical evidence that GGA may attenuate NSAID-induced intestinal inflammation relative to an active upper-gastrointestinal comparator.

At the transcriptomic level, one study [42] provided molecular-level mechanistic insights through microarray analysis of duodenal biopsies. *L. plantarum* CIP104448 significantly downregulated *GADD45B* expression (fold change, –1.223; *p* = 0.034), a stress-response gene involved in the glutathione-mediated detoxification pathway, suggesting modulation of cellular oxidative stress responses. The TIFN101 strain upregulated *GLUL*, which encodes glutamate-ammonia ligase (fold change, 1.155; *p* = 0.022), a key enzyme in glutamine biosynthesis and mucosal energy metabolism. Furthermore, the WCFS1 and CIP104448 strains upregulated *BRCA1* and *BRCA2* expression (*p* < 0.05), indicating activation of DNA repair mechanisms in NSAID-stressed duodenal epithelium. Collectively, these transcriptomic findings provide molecular-level support for the mechanistic synthesis presented in Section 4.4.

The remaining studies did not evaluate oxidative stress or inflammatory biomarkers. Nevertheless, several interventions associated with favorable or suggestive clinical effects were hypothesized to exert antioxidant or anti-inflammatory actions based on preclinical evidence cited by the respective authors (Table 4). These proposed mechanisms—including heat shock protein 70 (HSP70) induction [18,32,33], iron chelation [20], direct ROS scavenging [19,22], and provision of alternative energy substrates via nicotinamide adenine dinucleotide phosphate (NADPH) or bioactive peptides [21,23]—are broadly consistent with the oxidative and energetic stress cascades implicated in the pathogenesis of NSAID enteropathy.

## 4. Discussion

This systematic review synthesized evidence from 22 RCTs [18,19,20,21,22,23,24,25,30,31,32,33,34,35,36,37,38,39,40,41,42,43] evaluating the effects of natural products, functional foods, and probiotics on NSAID-induced small intestinal injury. The principal findings were as follows: (1) GGA demonstrated consistent mucosal protective effects in VCE studies [18,32,33]; (2) lactoferrin [20], zinc carnosine [22], and fish protein hydrolysate [21] each independently attenuated indomethacin-induced intestinal hyperpermeability in well-conducted crossover trials; (3) probiotic interventions demonstrated significant mucosal protective effects across all five VCE studies [24,25,38,39,40]; and (4) a striking outcome-dependent pattern was observed for probiotics, with no significant permeability effects in any of the three permeability studies [41,42,43], despite consistent efficacy in VCE-based assessments.

### 4.1. Geranylgeranylacetone

GGA is a terpenoid compound with well-established cytoprotective properties mediated through the induction of heat shock proteins (HSPs), particularly HSP70. These proteins stabilize cellular proteins under stress conditions and protect enterocytes from NSAID-induced mitochondrial uncoupling and subsequent apoptosis [8,44]. The consistent evidence from three out of four VCE studies [18,32,33] supports a biologically plausible protective role for GGA in the small intestine.

The negative finding reported in one pilot study [30] involving low-dose aspirin users may reflect the relatively lower severity of mucosal injury associated with this specific model compared with full-dose diclofenac studies. As proposed by the study authors, differences in the pathogenic mechanisms underlying aspirin versus non-aspirin NSAID-induced injury may also account for the variable efficacy of GGA. GGA is currently approved in Japan for gastric mucosal protection [45] and represents a natural-origin compound of significant clinical interest, given its established safety profile and targeted mechanism of action.

### 4.2. Polaprezinc

The within-group improvement in capsule endoscopy scores reported in one study [19] is consistent with the established mucosal protective effects of zinc carnosine complexes. These effects are mediated through the scavenging of ROS and inhibition of pro-apoptotic pathways, including the suppression of caspase-3 activation and mitochondrial cytochrome c release [46]. However, the absence of a between-group comparison limits the interpretability of these findings. Furthermore, this study [19] was rated as having some concerns overall, as the absence of a placebo control introduced potential performance bias despite the use of independently blinded endoscopic assessment. Consequently, adequately powered placebo-controlled trials are required to establish the efficacy of polaprezinc for this indication.

### 4.3. Lactoferrin, Zinc Carnosine, and Fish Protein Hydrolysate

The three independent crossover trials [20,21,22], demonstrating significant attenuation of indomethacin-induced intestinal hyperpermeability, provide convergent evidence for the barrier-protective potential of functionally diverse natural proteins and peptides. Lactoferrin exerts anti-inflammatory effects through the modulation of NF-κB signaling and the direct structural binding to NSAID molecules, thereby neutralizing their topical toxicity [47]. Experimental models have also shown that lactoferrin preserves tight junction integrity during NSAID-induced intestinal injury. Zinc carnosine exerts antioxidant and mucosal-stabilizing effects and has a well-established safety profile [22]. Fish protein hydrolysate contains bioactive peptides with growth-factor-like and barrier-protective activities [21,48]. Collectively, these findings suggest that enteral supplementation with natural barrier-protective proteins may represent a promising strategy for preventing NSAID-induced intestinal hyperpermeability, warranting further evaluation in larger, longer-duration trials.

### 4.4. Targeting the Oxidative Stress–Mitochondrial Injury Axis: Exploratory Mechanistic Considerations

A possible, but hypothesis-generating, mechanistic pattern suggested by this review is that several natural-origin interventions may be related to pathways involving oxidative stress, mitochondrial injury, inflammation, and epithelial repair. However, direct mechanistic biomarker evidence from the included clinical studies remains limited, as distinguished in Table 4. Most mechanistic interpretations, including those involving TLR4/NLRP3 signaling, IL-17A-mediated dysbiosis, and PPI-associated dysbiosis, are inferred from preclinical in vivo models, transcriptomic analyses, or mechanistic rationales cited by the original trial authors. Therefore, these pathways should be interpreted as possible mechanisms rather than established clinical explanations. The primary pathogenesis of NSAID-induced enteropathy involves the uncoupling of mitochondrial oxidative phosphorylation, leading to excessive ROS and intracellular ATP depletion. Together, these processes compromise epithelial tight junction integrity and increase paracellular permeability [8,49].

The interventions demonstrating favorable or suggestive effects in this review may counteract these pathways through distinct mechanisms that converge on oxidative stress and mitochondrial injury. For instance, lactoferrin [20,47] exerts its well-characterized iron-chelating activity that inhibits Fenton chemistry, thereby attenuating hydroxyl radical formation. Similarly, zinc carnosine [22,48] combines the free radical-scavenging properties of L-carnosine with the membrane-stabilizing effects of zinc, providing dual antioxidant and cytoprotective effects at the mucosal surface. Furthermore, glutamine [23] may provide an alternative energy source via NADPH generation, bypassing the NSAID-blocked ATP production pathway and buffering oxidative stress, whereas bioactive peptides in fish protein hydrolysate [21,48] may offer similar antioxidant and mucosal supportive benefits. Finally, GGA [18,32,33] may exert protective effects by inducing heat shock protein 70 (HSP70), which helps preserve mitochondrial function against uncoupling [44,45].

This interpretation is further supported by evidence that microbial metabolites, such as butyrate, can enhance intestinal barrier function and maintain mucosal homeostasis by modulating oxygen-sensing metabolic pathways [50], potentially providing a bioenergetic bridge that may help buffer ATP depletion triggered by NSAIDs. Mechanistic data from the included clinical studies also partially support this synthesis. One study [32] reported a significantly smaller post-treatment increase in fecal calprotectin in the GGA group compared with the famotidine group (between-group *p* = 0.041), providing indirect clinical evidence of anti-inflammatory activity beyond mucosal cytoprotection. Furthermore, transcriptomic data from another study [42] showed modest but statistically significant changes in genes related to glutamine biosynthesis and oxidative stress responses, including *GLUL* (fold change, 1.155; *p* = 0.022) and *GADD45B* (fold change, –1.223; *p* = 0.034), in probiotic-supplemented duodenal mucosa.

Collectively, this mechanistic synthesis suggests that natural compounds with antioxidant or energy-supportive properties may be biologically suited to attenuate key components of NSAID-induced small intestinal injury, although direct clinical biomarker evidence remains limited.

### 4.5. The Outcome-Dependent Pattern for Probiotics

A notable, but hypothesis-generating, observation of this review is the apparent divergence in reported probiotic and synbiotic findings between VCE and permeability studies. The available VCE-based studies generally reported favorable mucosal findings [24,25,38,39,40], whereas no clear effects on intestinal permeability were observed across the three permeability studies [41,42,43]. Several explanations for this apparent discordance merit consideration.

First, VCE and intestinal permeability tests assess fundamentally different pathophysiological constructs. VCE detects established mucosal lesions—erosions, ulcers, and mucosal breaks—that represent downstream manifestations of epithelial damage. In contrast, intestinal permeability assays reflect the functional integrity of tight junctions and the paracellular barrier at a mechanistic level. It is biologically plausible that probiotics primarily act by modulating the mucosal immune environment and reducing local inflammation, possibly through pathways involving TLR4/NLRP3 signaling [51] or by counteracting interleukin-17A-mediated dysbiosis [52], thereby promoting lesion healing or preventing progression of established injury, rather than directly preserving barrier function at the tight junction level in acute injury models.

Second, substantial methodological differences exist between the two sets of studies. Permeability trials were largely short-term crossover studies (5–14 days) conducted in healthy volunteers using indomethacin as the injury agent. In contrast, VCE studies involved relatively longer treatment periods (2–12 weeks) and were often conducted in patients already receiving aspirin for clinical indications. The shorter duration of permeability studies may be insufficient for meaningful microbiome modulation and probiotic colonization to exert detectable effects on barrier function. In addition, specific methodological limitations were noted in all three permeability studies, including a significant sex imbalance in one investigation [41] and potential tracer contamination in another [43].

Third, intestinal permeability assays themselves have recognized limitations, including sensitivity to a wide range of confounders and variable reproducibility. Consequently, biologically meaningful effects—such as the upregulation of DNA repair genes observed in one study [42]—may not be adequately captured by the relatively insensitive L/M or L/R ratio endpoints in the short-duration models employed.

Finally, the clinical impact of dysbiosis, particularly when induced by concurrent medications, must be addressed. Although PPIs are essential for upper gastrointestinal protection [53], they may exacerbate NSAID-induced small intestinal damage by altering the luminal microbiome [5]. This suggests a possible role for microbiome-modulating interventions in clinical settings where PPI-associated dysbiosis may exacerbate NSAID-induced inflammatory injury [54]. However, because the included probiotic and synbiotic studies differed substantially in strains, doses, exposure duration, background NSAID/aspirin use, and study populations, these findings should not be interpreted as evidence of a uniform class-wide probiotic effect. Any potential benefit is likely to be strain-specific, context-specific, and outcome-dependent.

### 4.6. Comparison with Existing Systematic Reviews

This review complements and extends a recent systematic review [3], which focused predominantly on synthetic pharmaceutical agents and concluded that the evidence supporting any single intervention was of low to moderate quality. By focusing specifically on natural products and probiotics and incorporating the CNKI database, the present review identifies a distinct body of evidence not captured by previous reviews. The additional Chinese-language publications, including the glutamine compound study [23], represent an important contribution from a region with substantial research activity in this field that remains underrepresented in major English-language databases.

### 4.7. Limitations

Several important limitations should be acknowledged. First, the overall certainty of evidence was low or very low, with only three of the 21 randomized trials [25,38,43] rated as having a low risk of bias, 15 rated as having some concerns, and three [24,31,33] rated as having a high risk of bias. The predominant methodological limitations across the included studies were the absence of placebo control in open-label designs, which introduced potential performance bias; the absence of prospective trial registration, which precluded definitive exclusion of selective outcome reporting; and incomplete reporting of allocation concealment procedures.

Although the narrative synthesis considered direction of effect, clinical context, study design, risk of bias, and consistency across studies, the absence of quantitative pooling limited our ability to compare effect sizes across interventions. In addition, because the included studies were generally small and short in duration, the safety profile of these interventions in the context of NSAID/aspirin-induced small intestinal injury should be considered reassuring but insufficiently characterized.

Second, the interventions, NSAID agents, study populations, and outcome measures were highly heterogeneous, precluding quantitative synthesis and limiting the strength of the overall conclusions drawn. Specifically, prevention studies in healthy volunteers with experimentally induced NSAID/aspirin injury and treatment-oriented studies in chronic aspirin/NSAID users with existing mucosal injury were interpreted as clinically distinct contexts. Findings from short-term prevention models should not be interpreted interchangeably with therapeutic efficacy in patients with established mucosal injury, which further limited our ability to draw unified conclusions. The broad intervention scope represents both a strength and a limitation: it enables a comprehensive overview of complementary and natural-origin strategies investigated for NSAID-induced small intestinal injury, but also restricts the ability to draw intervention-specific conclusions. Third, one eligible study available only as a conference abstract [39] was included due to sufficient extractable data, with this limitation transparently reported. Two additional conference abstracts were excluded because of insufficient data despite author contact. Fourth, CNKI searches, while adding important coverage of Chinese-language research, may have introduced challenges related to data verification. Fifth, publication bias cannot be ruled out.

Finally, although preclinical evidence suggests that oxidative stress is a central mediator of NSAID-induced enterocyte injury, most included studies assessed only structural or functional endpoints. Future clinical trials should incorporate a comprehensive panel of validated oxidative stress and inflammatory biomarkers—such as malondialdehyde, superoxide dismutase, and Nrf2 pathway-related markers [55]—to allow for more precise mechanistic inference alongside functional outcomes.

### 4.8. Clinical Implications and Future Research

The findings of this review suggest that several natural product interventions, particularly GGA, lactoferrin, zinc carnosine, and probiotics (based on VCE evidence), warrant further evaluation in adequately powered, methodologically rigorous RCTs. Future studies should employ standardized, validated outcome measures—ideally the Lewis score or mucosal break count via capsule endoscopy [56]—and adopt double-blind placebo-controlled designs. Head-to-head comparisons between natural products and established synthetic agents would be of particular clinical value. In addition, elucidation of the mechanisms underlying the observed outcome-dependent patterns for probiotics, including microbiome profiling, tight junction protein expression, and mucosal cytokine analysis, would significantly advance understanding in this field.

## 5. Conclusions

This systematic review synthesized evidence from 22 studies evaluating natural products, functional foods, probiotics, and synbiotics for NSAID/aspirin-induced small intestinal injury. Preliminary evidence from independent trials suggests that selected natural-origin interventions, particularly GGA, lactoferrin, zinc carnosine, and fish protein hydrolysate, may have protective effects. Probiotic and synbiotic interventions showed an outcome-dependent pattern, with more consistent favorable findings for capsule endoscopy-based mucosal injury outcomes than for intestinal permeability outcomes. However, given the low or very low certainty of evidence and substantial clinical and methodological heterogeneity, these findings should be interpreted as hypothesis-generating rather than definitive.

Given the unmet clinical need for effective preventive strategies against NSAID/aspirin-induced small intestinal injury, further high-quality RCTs are warranted. Future studies should incorporate standardized outcome measures, double-blind placebo-controlled designs, adequate sample sizes, and sufficient safety monitoring. In addition, direct measurement of oxidative stress, inflammatory, epithelial barrier, and microbiome-related biomarkers may help clarify the potential mechanisms underlying these preliminary findings.

## Figures and Tables

**Table 1:** Characteristics of included studies.

Author, Year	Country	Intervention	NSAID	Design	*N*	Duration	Outcome	Purpose	Key Result
Niwa et al. [18]	Japan	GGA 300 mg/day	Diclofenac	RCT, DB, crossover (2-week washout; combined data)	40	2 weeks	VCE	Prevention	Gastric + SI combined score, *p* = 0.027 (SI alone NS)
Shiotani et al. [30]	Japan	GGA 150 mg/day	Aspirin	RCT, parallel, DB	42	8 weeks	VCE	Prevention	NS (low-dose aspirin model)
Watari et al. [19]	Japan	Polaprezinc 150 mg/day	Aspirin	RCT, parallel, open-label	36	4 weeks	VCE	Treatment	Within-group erosion reduction, *p* = 0.039 (no between-group comparison)
Huang et al. [31]	China	Muscovite 3 g × 2/day	Diclofenac	RCT, parallel, open-label	21	2 weeks	VCE	Prevention	Mucosal breaks 31.3% vs. 71.4%, *p* = 0.028
Umegaki et al. [32]	Japan	GGA 150 mg/day	Diclofenac	RCT, parallel, DB	42	2 weeks	VCE	Prevention	Erythema, *p* = 0.032; ulcer, *p* = 0.017 (vs. Famotidine)
Xiong et al. [33]	China	GGA 150 mg/day	Diclofenac	RCT, parallel, DB	60	12 weeks	VCE	Prevention	Post-treatment Lewis score lower, *p* = 0.017
Chen, Lang & Zhang [34]	China	Glutamine compound + herbal	Aspirin	RCT, parallel, open-label	57	8 weeks	VCE	Treatment	Injury score reduction, *p* < 0.001; erosion improvement 78.9% vs. 5.6%
Hond et al. [23]	Belgium	Glutamine (multiple doses)	Indomethacin	Quasi-RCT, crossover (washout not clearly reported)	14	1 week	L/M ratio	Prevention	Multiple-dose co-admin: permeability reduced vs. NSAID alone (*p* = 0.001); pre-treatment and single-dose: NS (ROBINS-I: Serious; excluded from primary analysis)
Troost, Saris & Brummer [20]	The Netherlands	Lactoferrin 5 g/day	Indomethacin	RCT, DB, crossover (2-week washout; combined data)	12	7 days	L/R ratio	Prevention	L/R 0.036 → 0.028, *p* < 0.05
Bours [35]	The Netherlands	ATP enteric capsule	Indomethacin	RCT, DB, crossover (2-week washout; combined data)	12	5 days	L/R ratio	Prevention	NS (oral route, site mismatch)
Bours [36]	The Netherlands	ATP naso-jejunal infusion	Indomethacin	RCT, DB, crossover (1-week washout; combined data)	12	5 days	L/R ratio	Prevention	L/R 0.042 → 0.027, *p* < 0.01
Mahmood et al. [22]	UK	Zinc carnosine 37.5 mg × 2/day	Indomethacin	RCT, DB, crossover (2-week washout; combined data)	10	5 days	L/M ratio	Prevention	Placebo: threefold increase in L:R ratio (*p* < 0.01); ZnC: no significant increase
Marchbank et al. [21]	UK	Fish protein hydrolysate 1 g × 3/day	Indomethacin	RCT, crossover (2-week washout; combined data)	10	5 days	L/M ratio	Prevention	0.35 → 0.59 vs. 0.28 → 1.54, *p* < 0.01
Ganda Mall et al. [37]	Sweden	AX or OBG 12 g/day	Indomethacin	RCT, parallel, DB	49	6 weeks	L/M ratio	Prevention	NS (elderly population; reduced colonic fermentation capacity suggested as explanation)
Endo et al. [24]	Japan	*L. casei* Shirota	Aspirin 100 mg/day	RCT, parallel, open-label	41	12 weeks	VCE	Treatment	CE score, *p* = 0.026
Suzuki [38]	Japan	*L. gasseri* fermented milk	Aspirin	RCT, parallel, DB	36	6 weeks	VCE	Treatment	Mucosal breaks, *p* < 0.01
Mortensen et al. [25]	Denmark	*B. breve* Bif195	Aspirin 300 mg/day	RCT, parallel, DB	22	2 weeks	VCE	Prevention	Lewis AUC 3040 vs. 4351, *p* = 0.038
Okamoto et al. [39]	Japan	*L. salivarius* WB21	Celecoxib + Rabeprazole	RCT, parallel, DB	57	2 weeks	VCE	Prevention	Ulcer incidence 14.8% vs. 40%, *p* = 0.043
Chen [40]	China	Lactobacillus complex (4 strains)	Aspirin 100 mg/day	RCT, parallel, open-label	60	8 weeks	VCE	Treatment	Injury score, *p* < 0.001
Wilms [41]	The Netherlands	Ecologic 825 + FOS P6	Indomethacin	RCT, parallel, DB	20	2 weeks	L/M ratio	Prevention	NS (sex imbalance noted)
Mujagic [42]	The Netherlands	*L. plantarum* (3 strains)	Indomethacin	RCT, DB, crossover (4-week washout; combined data)	10	7 days	L/M ratio	Prevention	NS (gene expression only partially significant)
Judkins [43]	USA	*L. helveticus* R0052 + *L. rhamnosus* R0011	Aspirin 1950 mg (acute)	RCT, DB, crossover (4-week washout; combined data)	29	3 weeks	L/M ratio	Prevention	NS (L-rhamnose → neotame contamination noted)

DB, double-blind; *N*, number of participants; GGA, geranylgeranylacetone; L/M, lactulose–mannitol ratio; L/R, lactulose–rhamnose ratio; NS, not significant; RCT, randomized controlled trial; VCE, video capsule endoscopy; ATP, adenosine triphosphate; NSAID, nonsteroidal anti-inflammatory drug; ROBINS-I, Risk of Bias in Non-randomized Studies of Interventions; AUC, area under the curve; SI, small intestine.

**Table 2:** Summary of findings across included studies.

Intervention	Outcome	*N*	NSAID	Design	Key Findings	Overall RoB
Group A: Natural Products and Functional Foods—VCE subgroup						
GGA	VCE	4	Diclofenac (3); Aspirin (1)	3 RCT, DB; 1 open-label	Significant in 3/4 studies; NS in low-dose aspirin model [30]	3× some concerns; 1× high
Polaprezinc	VCE	1	Aspirin	RCT, open-label	Within-group erosion reduction, *p* = 0.039; no between-group comparison	Some concerns
Muscovite	VCE	1	Diclofenac	RCT, open-label	Mucosal breaks 31.3% vs. 71.4%, *p* = 0.028	High
Glutamine + herbal compound	VCE	1	Aspirin	RCT, open-label	Injury score, *p* < 0.001; erosion improvement 78.9% vs. 5.6%	Some concerns
Group A: Natural Products and Functional Foods—Permeability subgroup						
Lactoferrin	Permeability	1	Indomethacin	RCT crossover, DB	L/R ratio reduced vs. placebo, *p* < 0.05	Some concerns
ATP (naso-jejunal infusion)	Permeability	1	Indomethacin	RCT crossover, DB	L/R 0.042 → 0.027, *p* < 0.01	Some concerns
ATP (oral enteric capsule)	Permeability	1	Indomethacin	RCT crossover, DB	NS (delivery site mismatch)	Some concerns
Zinc carnosine	Permeability	1	Indomethacin	RCT crossover	Placebo: ~3-fold L:R increase (*p* < 0.01); ZnC: no significant increase	Some concerns
Fish protein hydrolysate	Permeability	1	Indomethacin	RCT crossover	L/M 0.35 → 0.59 vs. 0.28 → 1.54, *p* < 0.01	Some concerns
Glutamine (multiple dose)	Permeability	1	Indomethacin	Quasi-RCT crossover	Simultaneous co-admin: *p* = 0.001; pre-treatment/single dose: NS	ROBINS-I: serious
Dietary fiber (AX or OBG, 12 g/day)	Permeability	1	Indomethacin	RCT, parallel, DB	NS (elderly population)	Some concerns
Group B: Probiotics and Synbiotics—VCE subgroup						
*L. casei* Shirota	VCE	1	Aspirin	RCT, parallel, open-label	CE score, *p* = 0.026	High
*L. gasseri* (fermented milk)	VCE	1	Aspirin	RCT, parallel, DB	Mucosal breaks, *p* < 0.01	Low
*B. breve* Bif195	VCE	1	Aspirin 300 mg/day	RCT, parallel, DB	Lewis AUC 3040 vs. 4351, *p* = 0.038	Low
*L. salivarius* WB21	VCE	1	Celecoxib + PPI	RCT, DB	Ulcer incidence 14.8% vs. 40.0%, *p* = 0.043	Some concerns
Lactobacillus complex (4 strains)	VCE	1	Aspirin	RCT, open-label	Injury score, *p* < 0.001	Some concerns
Group B: Probiotics and Synbiotics—Permeability subgroup						
Synbiotic (Ecologic 825 + FOS P6)	Permeability	1	Indomethacin	RCT, parallel, DB	NS (sex imbalance between groups)	Some concerns
*L. plantarum* (3 strains)	Permeability	1	Indomethacin	RCT crossover, DB	NS (gene expression partially modulated)	Some concerns
*L. helveticus* R0052 + *L. rhamnosus* R0011	Permeability	1	Aspirin 1950 mg (acute)	RCT crossover, DB	NS (tracer substitution noted)	Low

AX, arabinoxylan; OBG, oat β-glucan; PPI, proton pump inhibitor; RoB, risk of bias—Hond et al. [23] assessed by ROBINS-I (not RoB 2). ROB ratings reflect overall domain-level assessment per the Cochrane RoB 2 tool.

**Table 3:** Summary of findings: GRADE-informed certainty of evidence by intervention category and outcome domain.

Intervention Category	Outcome Domain	No. of Studies	Summary of Findings	Certainty of Evidence	Main Reasons for Limited Certainty
Geranylgeranylacetone (GGA)	Capsule endoscopy-based mucosal injury	4	Several studies reported favorable findings, although results varied across NSAID/aspirin models and outcome definitions.	Low	Risk of bias, inconsistency, imprecision, small sample sizes
Other natural-origin interventions	Capsule endoscopy-based mucosal injury	3	Suggestive findings were reported for polaprezinc, muscovite, and a glutamine-containing compound preparation, but interpretation was limited by open-label designs, within-group-only findings, or small samples.	Very low	Risk of bias, indirectness, imprecision, intervention heterogeneity
Individual natural-origin compounds/components	Intestinal permeability	3	Three small independent crossover trials, each evaluating a distinct intervention, reported favorable findings for lactoferrin, zinc carnosine, or fish protein hydrolysate.	Low	Small sample sizes, imprecision, indirectness, intervention heterogeneity
Other natural-origin interventions	Intestinal permeability	4	Findings were mixed across ATP formulations, dietary fiber, and glutamine. One quasi-randomized glutamine study was retained as supportive evidence only.	Very low	Risk of bias, heterogeneity, imprecision, indirectness
Probiotics/synbiotics	Capsule endoscopy-based mucosal injury	5	All studies reported favorable capsule endoscopy-based findings, but interpretation should be strain- and context-specific rather than considered evidence of a uniform probiotic class effect.	Low	Strain heterogeneity, risk of bias, imprecision, differences in populations and NSAID/aspirin exposure
Probiotics/synbiotics	Intestinal permeability	3	No clear effect on permeability outcomes was observed across three small studies.	Very low	Small number of studies, imprecision, heterogeneity, short intervention duration

Certainty ratings were informed by GRADE domains, including risk of bias, inconsistency, indirectness, imprecision, and publication bias. Because quantitative pooling was not performed, ratings were based on a narrative assessment of the overall body of evidence within each intervention category and outcome domain rather than pooled effect estimates. One quasi-randomized study (Den Hond et al., 1999 [23]) was assessed separately using ROBINS-I and considered supportive evidence only. GGA: geranylgeranylacetone; NSAID: nonsteroidal anti-inflammatory drug; RoB: risk of bias.

**Table 4:** Mechanistic correlates related to oxidative stress, inflammation, and cellular repair across included studies.

Study	Intervention	Biomarker/Target	Mechanistic Outcome	Biological Implication
Section 1. Direct Biomarker Measurements				
Ganda Mall et al. [37]	OBG (12 g/day)	Plasma H_2_O_2_ (FORT assay)	Significant reduction (*p* = 0.0005)	Attenuation of systemic oxidative stress (ROS)
Mortensen et al. [25]	*B. breve* Bif195	Fecal calprotectin AUC	Significant reduction vs. placebo (*p* = 0.0347)	Suppression of intestinal mucosal inflammation
Umegaki et al. [32]	GGA	Fecal calprotectin	The GGA group showed significantly smaller post-treatment calprotectin increase vs. famotidine (between-group *p* = 0.041); neither group showed a significant within-group increase	Relative attenuation of NSAID-induced intestinal inflammation vs. active comparator
Section 2. Transcriptomic Evidence				
Mujagic et al. [42]	*L. plantarum* CIP104448	GADD45B gene	Downregulation (FC, –1.223; *p* = 0.034)	Modulation of the glutathione-mediated detoxification pathway
Mujagic et al. [42]	*L. plantarum* TIFN101	GLUL gene	Upregulation (FC, 1.155, *p* = 0.022)	Enhancement of glutamine biosynthesis and mucosal cell energy supply
Mujagic et al. [42]	*L. plantarum* WCFS1/CIP104448	BRCA1/BRCA2 genes	Upregulation (*p* < 0.05)	Activation of DNA repair in NSAID-stressed duodenal epithelium
Section 3. Proposed Mechanisms (Preclinical Evidence Cited by Trial Authors) *				
Niwa et al. [18]; Umegaki et al. [32]; Xiong et al. [33]	GGA	HSP70	Induction of HSP70	Mitochondrial protection against NSAID-induced uncoupling stress
Troost et al. [20]	Lactoferrin	Free iron/hydroxyl radical	Iron chelation inhibiting Fenton chemistry	Prevention of hydroxyl radical generation and oxidative mucosal injury
Watari et al. [19]; Mahmood et al. [22]	Zinc carnosine/Polaprezinc	ROS	Direct free radical scavenging by L-carnosine and zinc	Cytoprotection and prevention of oxidative apoptosis in enterocytes
Hond et al. [23]	Glutamine	NADPH/ATP pathway	NADPH generation bypassing NSAID-blocked oxidative phosphorylation	Alternative energy supply buffering ATP depletion and oxidative stress
Marchbank et al. [21]	Fish protein hydrolysate	Bioactive peptides (glutamine-rich)	Barrier-protective and mucosal repair activity via bioactive peptide components	Antioxidant support and mucosal structural repair

* Proposed mechanisms reflect the theoretical rationale explicitly stated by the authors of the respective clinical trials, based on pre-clinical models. FORT, Free Oxygen Radicals Test; GADD45B, growth arrest and DNA damage-inducible beta; GLUL, glutamate-ammonia ligase; HSP70, heat shock protein 70; ROS, reactive oxygen species; H_2_O_2_, hydrogen peroxide; NADPH, nicotinamide adenine dinucleotide phosphate; FC, fold change.

## Data Availability

No new data were created or analyzed in this study. Data sharing is not applicable to this article.

## Supplementary Materials

The following supporting information can be downloaded at: https://www.mdpi.com/article/10.3390/antiox15070903/s1, Table S1: Full Electronic Search Strategies.

## Abbreviations

The following abbreviations are used in this manuscript:

ATP	Adenosine Triphosphate
AUC	Area Under the Curve
CENTRAL	Cochrane Central Register of Controlled Trials
CNKI	China National Knowledge Infrastructure
GGA	Geranylgeranylacetone
GLUL	Glutamate-Ammonia Ligase
HSP	Heat Shock Protein
HSP70	Heat Shock Protein 70
ICTRP	International Clinical Trials Registry Platform
L/M Ratio	Lactulose-to-Mannitol Ratio
L/R Ratio	Lactulose-to-Rhamnose Ratio
MCCE	Magnetically Controlled Capsule Endoscopy
NADPH	Nicotinamide Adenine Dinucleotide Phosphate
NSAIDs	Nonsteroidal Anti-Inflammatory Drugs
PPI	Proton Pump Inhibitor
PRISMA	Preferred Reporting Items for Systematic Reviews and Meta-Analyses
RCT	Randomized Controlled Trial
RoB 2	Risk of Bias 2
ROBINS-I	Risk Of Bias In Non-randomized Studies of Interventions
ROS	Reactive Oxygen Species
SWiM	Synthesis Without Meta-analysis
VCE	Video Capsule Endoscopy
WHO	World Health Organization

## References

[1] Scarpignato C., Hunt R.H. (2010). Nonsteroidal antiinflammatory drug-related injury to the gastrointestinal tract: Clinical picture, pathogenesis, and prevention. Gastroenterol. Clin. N. Am..

[2] Zhang M., Xia F., Xia S., Zhou W., Zhang Y., Han X., Zhao K., Feng L., Dong R., Tian D. (2022). NSAID-associated small intestinal injury: An overview from animal model development to pathogenesis, treatment, and prevention. Front. Pharmacol..

[3] Choe Y., Park J.M., Kim J.S., Cho Y.K., Kim B.W., Choi M.G., Kim N.J. (2024). Drugs effective for nonsteroidal anti-inflammatory drugs or aspirin-induced small bowel injuries: A systematic review and meta-analysis of randomized controlled trials. J. Clin. Gastroenterol..

[4] Bjarnason I., Scarpignato C., Holmgren E., Olszewski M., Rainsford K.D., Lanas A. (2018). Mechanisms of damage to the gastrointestinal tract from nonsteroidal anti-inflammatory drugs. Gastroenterology.

[5] Washio E., Esaki M., Maehata Y., Miyazaki M., Kobayashi H., Ishikawa H., Kitazono T., Matsumoto T. (2016). Proton pump inhibitors increase incidence of nonsteroidal anti-inflammatory drug–induced small bowel injury: A randomized, placebo-controlled trial. Clin. Gastroenterol. Hepatol..

[6] Maiden L., Thjodleifsson B., Theodors A., Gonzalez J., Bjarnason I. (2005). A quantitative analysis of NSAID-induced small bowel pathology by capsule enteroscopy. Gastroenterology.

[7] Watanabe T., Fujiwara Y., Chan F.K.L. (2020). Current knowledge on non-steroidal anti-inflammatory drug-induced small-bowel damage: A comprehensive review. J. Gastroenterol..

[8] Somasundaram S., Rafi S., Hayllar J., Sigthorsson G., Jacob M., Price A.B., Macpherson A., Mahmod T., Scott D., Wrigglesworth J.M. (1997). Mitochondrial Damage: A Possible Mechanism of the “topical” Phase of NSAID Induced Injury to the Rat Intestine. Gut.

[9] Handa O., Majima A., Onozawa Y., Horie H., Uehara Y., Fukui A., Omatsu T., Naito Y., Yoshikawa T. (2014). The role of mitochondria-derived reactive oxygen species in the pathogenesis of non-steroidal anti-inflammatory drug-induced small intestinal injury. Free Radic. Res..

[10] Reuter B.K., Davies N.M., Wallace J.L. (1997). Nonsteroidal anti-inflammatory drug enteropathy in rats: Role of permeability, bacteria, and enterohepatic circulation. Gastroenterology.

[11] Ma Y., Yin Z., Li L., Chen B., Dai H., Wu D., Cong J., Ye L., Liao C., Li L. (2021). Food antigens exacerbate intestinal damage and inflammation following the disruption of the mucosal barrier. Int. Immunopharmacol..

[12] Lanas A., Sopeña F. (2009). Nonsteroidal anti-inflammatory drugs and lower gastrointestinal complications. Gastroenterol. Clin. N. Am..

[13] Wallace J.L., Syer S., Denou E., de Palma G., Vong L., McKnight W., Jury J., Bolla M., Bercik P., Collins S.M. (2011). Proton pump inhibitors exacerbate NSAID-induced small intestinal injury by inducing dysbiosis. Gastroenterology.

[14] Kyaw M.H., Otani K., Ching J.Y.L., Higashimori A., Kee K.M., Watanabe T., Tse Y.K., Lee V., Tanigawa T., Cheong P.K. (2018). Misoprostol heals small bowel ulcers in aspirin users with small bowel bleeding. Gastroenterology.

[15] Taha A.S., McCloskey C., McSkimming P., McConnachie A. (2018). Misoprostol for small bowel ulcers in patients with obscure bleeding taking aspirin and non-steroidal anti-inflammatory drugs (MASTERS): A randomised, double-blind, placebo-controlled, phase 3 trial. Lancet Gastroenterol. Hepatol..

[16] Scarpignato C., Dolak W., Lanas A., Matzneller P., Renzulli C., Grimaldi M., Zeitlinger M., Bjarnason I. (2017). Rifaximin reduces the number and severity of intestinal lesions associated with use of nonsteroidal anti-inflammatory drugs in humans. Gastroenterology.

[17] Kurokawa S., Katsuki S., Fujita T., Saitoh Y., Ohta H., Nishikawa K., Sato Y., Sato Y., Ohira K., Yamada M. (2014). A randomized, double-blinded, placebo-controlled, multicenter trial, healing effect of rebamipide in patients with low-dose aspirin and/or non-steroidal anti-inflammatory drug induced small bowel injury. J. Gastroenterol..

[18] Niwa Y., Nakamura M., Miyahara R., Ohmiya N., Watanabe O., Ando T., Kawashima H., Itoh A., Hirooka Y., Goto H. (2009). Geranylgeranylacetone protects against diclofenac-induced gastric and small intestinal mucosal injuries in healthy subjects: A prospective randomized placebo-controlled double-blind cross-over study. Digestion.

[19] Watari I., Oka S., Tanaka S., Aoyama T., Imagawa H., Shishido T., Yoshida S., Chayama K. (2013). Effectiveness of polaprezinc for low-dose aspirin-induced small-bowel mucosal injuries as evaluated by capsule endoscopy: A pilot randomized controlled study. BMC Gastroenterol..

[20] Troost F.J., Saris W.H.M., Brummer R.J.M. (2003). Recombinant human lactoferrin ingestion attenuates indomethacin-induced enteropathy in vivo in healthy volunteers. Eur. J. Clin. Nutr..

[21] Marchbank T., Limdi J.K., Mahmood A., Elia G., Playford R.J. (2008). Clinical trial: Protective effect of a commercial fish protein hydrolysate against indomethacin (NSAID)-induced small intestinal injury. Aliment. Pharmacol. Ther..

[22] Mahmood A., FitzGerald A.J., Marchbank T., Ntatsaki E., Murray D., Ghosh S., Playford R.J. (2007). Zinc carnosine, a health food supplement that stabilises small bowel integrity and stimulates gut repair processes. Gut.

[23] Hond E.D., Peeters M., Hiele M., Bulteel V., Ghoos Y., Rutgeerts P. (1999). Effect of glutamine on the intestinal permeability changes induced by indomethacin in humans. Aliment. Pharmacol. Ther..

[24] Endo H., Higurashi T., Hosono K., Sakai E., Sekino Y., Iida H., Sakamoto Y., Koide T., Takahashi H., Yoneda M. (2011). Efficacy of *Lactobacillus casei* treatment on small bowel injury in chronic low-dose aspirin users: A pilot randomized controlled study. J. Gastroenterol..

[25] Mortensen B., Murphy C., O’Grady J., Lucey M., Elsafi G., Barry L., Westphal V., Wellejus A., Lukjancenko O., Eklund A.C. (2019). Bifidobacterium breve Bif195 protects against small-intestinal damage caused by acetylsalicylic acid in healthy volunteers. Gastroenterology.

[26] Page M.J., McKenzie J.E., Bossuyt P.M., Boutron I., Hoffmann T.C., Mulrow C.D., Shamseer L., Tetzlaff J.M., Akl E.A., Brennan S.E. (2021). The PRISMA 2020 statement: An updated guideline for reporting systematic reviews. BMJ.

[27] Sterne J.A.C., Hernán M.A., Reeves B.C., Savović J., Berkman N.D., Viswanathan M., Henry D., Altman D.G., Ansari M.T., Boutron I. (2016). ROBINS-I: A risk of bias instrument for non-randomized studies of interventions. BMJ.

[28] Sterne J.A.C., Savović J., Page M.J., Elbers R.G., Blencowe N.S., Boutron I., Cates C.J., Cheng H.Y., Corbett M.S., Eldridge S.M. (2019). RoB 2: A revised tool for assessing risk of bias in randomised trials. BMJ.

[29] Campbell M., McKenzie J.E., Sowden A., Katikireddi S.V., Brennan S.E., Ellis S., Hartmann-Boyce J., Ryan R., Shepperd S., Thomas J. (2020). Synthesis without meta-analysis (SWiM) in systematic reviews: Reporting guideline. BMJ.

[30] Shiotani A., Haruma K., Nishi R., Fujita M., Kamada T., Honda K., Kusunoki H., Hata J., Graham D.Y. (2010). Randomized, double-blind, pilot study of geranylgeranylacetone versus placebo in patients taking low-dose enteric-coated aspirin. Low-dose aspirin-induced small bowel damage. Scand. J. Gastroenterol..

[31] Huang C. (2014). Muscovite is protective against non-steroidal anti-inflammatory drug-induced small bowel injury. World J. Gastroenterol..

[32] Umegaki E., Kuramoto T., Kojima Y., Nouda S., Ishida K., Takeuchi T., Inoue T., Tokioka S., Higuchi K. (2014). Geranylgeranylacetone, a gastromucoprotective drug, protects against NSAID-induced esophageal, gastroduodenal and small intestinal mucosal injury in healthy subjects: A prospective randomized study involving a comparison with famotidine. Intern. Med..

[33] Xiong L., Huang X., Li L., Yang X., Liang L., Zhan Z., Ye Y., Chen M. (2015). Geranylgeranylacetone protects against small-intestinal injuries induced by diclofenac in patients with rheumatic diseases: A prospective randomized study. Dig. Liver Dis..

[34] Chen X., Lang H., Zhang J. (2024). A randomized controlled trial of glutamine in the treatment of small intestinal mucosal injury in patients taking aspirin. China Med..

[35] Bours M.J.L., Bos H.J., Meddings J.B., Brummer R.J.M., van den Brandt P.A., Dagnelie P.C. (2007). Effects of oral adenosine 5′-triphosphate and adenosine in enteric-coated capsules on indomethacin-induced permeability changes in the human small intestine: A randomized cross-over study. BMC Gastroenterol..

[36] Bours M.J.L., Troost F.J., Brummer R.J.M., Bast A., Dagnelie P.C. (2007). Local effect of adenosine 5′-triphosphate on indomethacin-induced permeability changes in the human small intestine. Eur. J. Gastroenterol. Hepatol..

[37] Ganda Mall J.-P., Fart F., Sabet J.A., Lindqvist C.M., Nestestog R., Hegge F.T., Keita Å.V., Brummer R.J., Schoultz I. (2020). Effects of dietary fibres on acute indomethacin-induced intestinal hyperpermeability in the elderly: A randomised placebo controlled parallel clinical trial. Nutrients.

[38] Suzuki T., Masui A., Nakamura J., Shiozawa H., Aoki J., Nakae H., Tsuda S., Imai J., Hideki O., Matsushima M. (2017). Yogurt containing *Lactobacillus gasseri* mitigates aspirin-induced small bowel injuries: A prospective, randomized, double-blind, placebo-controlled trial. Digestion.

[39] Okamoto Y., Esaki M., Morishita T., Hara Y., Hirano A., Umeno J., Maehata Y., Kobayashi H., Ishikawa H., Torisu T. (2019). Preventive effect of *Lactobacillus salivarius* WB21 on small bowel injuries in subjects who take both NSAID and PPI: A randomized, double-blind, placebo-controlled trial. Presented at: 27th United European Gastroenterology Week, UEG.. U Eur. Gastroenterol. J..

[40] Chen X., Gao F., Zhang J. (2022). Lactobacillus complex capsules ameliorate aspirin-related small intestinal mucosal injury: A prospective, randomized, controlled clinical trial. Scand. J. Gastroenterol..

[41] Wilms E., Gerritsen J., Smidt H., Besseling-van der Vaart I., Rijkers G.T., Garcia Fuentes A.R., Masclee A.A.M., Troost F.J. (2016). Effects of supplementation of the synbiotic Ecologic^®^ 825/FOS P6 on intestinal barrier function in healthy humans: A randomized controlled trial. PLoS ONE.

[42] Mujagic Z., de Vos P., Boekschoten M.V., Govers C., Pieters H.J.H.M., de Wit N.J.W., Bron P.A., Masclee A.A.M., Troost F.J. (2017). The effects of Lactobacillus plantarum on small intestinal barrier function and mucosal gene transcription; a randomized double-blind placebo controlled trial. Sci. Rep..

[43] Judkins T.C., Solch-Ottaiano R.J., Ceretto-Clark B., Nieves C., Colee J., Wang Y., Tompkins T.A., Caballero-Calero S.E., Langkamp-Henken B. (2024). The effect of an acute aspirin challenge on intestinal permeability in healthy adults with and without prophylactic probiotic consumption: A double-blind, placebo-controlled, randomized trial. BMC Gastroenterol..

[44] Yanaka A., Zhang S., Sato D., Tauchi M., Suzuki H., Shibahara T., Matsui H., Nakahara A., Hyodo I. (2007). Geranylgeranylacetone protects the human gastric mucosa from diclofenac-induced injury via induction of heat shock protein 70. Digestion.

[45] Rokutan K. (2000). Role of heat shock proteins in gastric mucosal protection. J. Gastroenterol. Hepatol..

[46] Omatsu T., Naito Y., Handa O., Mizushima K., Hayashi N., Qin Y., Harusato A., Hirata I., Kishimoto E., Okada H. (2010). Reactive oxygen species-quenching and anti-apoptotic effect of polaprezinc on indomethacin-induced small intestinal epithelial cell injury. J. Gastroenterol..

[47] Mir R., Singh N., Vikram G., Kumar R.P., Sinha M., Bhushan A., Kaur P., Srinivasan A., Sharma S., Singh T.P. (2009). The structural basis for the prevention of nonsteroidal antiinflammatory drug-induced gastrointestinal tract damage by the C-lobe of bovine colostrum lactoferrin. Biophys. J..

[48] Playford R.J. (1995). Peptides and gastrointestinal mucosal integrity. Gut.

[49] Takeuchi K., Satoh H. (2015). NSAID-induced small intestinal damage—Roles of various pathogenic factors. Digestion.

[50] Fachi J.L., Felipe J.d.S., Pral L.P., da Silva B.K., Corrêa R.O., de Andrade M.C.P., da Fonseca D.M., Basso P.J., Câmara N.O.S., de Sales e Souza É.L. (2019). Butyrate protects mice from Clostridium difficile-induced colitis through an HIF-1-Dependent mechanism. Cell Rep..

[51] Otani K., Tanigawa T., Watanabe T., Shimada S., Nadatani Y., Nagami Y., Tanaka F., Kamata N., Yamagami H., Shiba M. (2017). Microbiota plays a key role in non-steroidal anti-inflammatory drug-induced small intestinal damage. Digestion.

[52] Sugimura N., Otani K., Watanabe T., Nakatsu G., Shimada S., Fujimoto K., Nadatani Y., Hosomi S., Tanaka F., Kamata N. (2019). High-fat diet-mediated dysbiosis exacerbates NSAID-induced small intestinal damage through the induction of interleukin-17A. Sci. Rep..

[53] Scarpignato C., Gatta L., Zullo A., Blandizzi C. (2016). Effective and safe proton pump inhibitor therapy in acid-related diseases—A position paper addressing benefits and potential harms of acid suppression. BMC Med..

[54] Maseda D., Ricciotti E. (2020). NSAID-gut microbiota interactions. Front. Pharmacol..

[55] Ma Q. (2013). Role of nrf2 in oxidative stress and toxicity. Annu. Rev. Pharmacol. Toxicol..

[56] Yablecovitch D., Lahat A., Neuman S., Levhar N., Avidan B., Ben-Horin S., Eliakim R., Kopylov U. (2018). The Lewis score or the capsule endoscopy Crohn’s disease activity index: Which one is better for the assessment of small bowel inflammation in established Crohn’s disease?. Ther. Adv. Gastroenterol..

