# Supplementary material for: Protective Effects of Natural Products, Functional Foods, and Probiotics on NSAID-Induced Small Intestinal Injury: A Systematic Review with Mechanistic Considerations of Oxidative Stress and Microbiome Modulation
Source: Antioxidants (Basel). 2026 Jul 21;15(7):903. doi: 10.3390/antiox15070903 (PMC13405968; doi:10.3390/antiox15070903)
Supplement: Supplementary file 1 [file antioxidants-15-00903-s001.zip › antioxidants-4369344-supplementary.pdf]

Table S1. Full Electronic Search Strategies

Database	Search Strategy	Records Retrieved
PubMed	#1 NSAID*[tiab] OR "non-steroidal anti-inflammatory"[tiab] OR "nonsteroidal anti-inflammatory"[tiab] OR aspirin[tiab] OR indomethacin[tiab] OR diclofenac[tiab] OR naproxen[tiab] OR ibuprofen[tiab] OR celecoxib[tiab] OR loxoprofen[tiab]<br>#2 "small intestin*"[tiab] OR "small bowel"[tiab] OR jejun*[tiab] OR ileum[tiab] OR ileal[tiab] OR enteropathy[tiab] OR enteropathies[tiab]<br>#3 "mucosal injur*"[tiab] OR "intestinal injur*"[tiab] OR "intestinal damage"[tiab] OR "intestinal permeab*"[tiab] OR "capsule endoscop*"[tiab] OR "video capsule"[tiab] OR erosion*[tiab] OR ulcer*[tiab] OR "Lewis score"[tiab] OR lactulose[tiab] OR mannitol[tiab]<br>#4 "randomized controlled trial"[pt] OR randomized[tiab] OR randomised[tiab] OR placebo[tiab] OR "double-blind"[tiab] OR crossover[tiab] OR "cross-over"[tiab]<br>#5 #1 AND #2 AND #3 AND #4	96 records
Embase	#1 NSAID*:ab,ti OR 'non-steroidal anti-inflammatory':ab,ti OR 'nonsteroidal anti-inflammatory':ab,ti OR aspirin:ab,ti OR indomethacin:ab,ti OR diclofenac:ab,ti OR naproxen:ab,ti OR ibuprofen:ab,ti OR celecoxib:ab,ti OR loxoprofen:ab,ti OR 'low-dose aspirin':ab,ti<br>#2 'small intestin*':ab,ti OR 'small bowel':ab,ti OR jejun*:ab,ti OR ileum:ab,ti OR ileal:ab,ti OR enteropathy:ab,ti OR enteropathies:ab,ti<br>#3 'mucosal injur*':ab,ti OR 'intestinal injur*':ab,ti OR 'intestinal damage':ab,ti OR 'intestinal permeab*':ab,ti OR 'capsule endoscop*':ab,ti OR 'video capsule':ab,ti OR erosion*:ab,ti OR ulcer*:ab,ti OR 'lewis score':ab,ti OR lactulose:ab,ti OR mannitol:ab,ti<br>#4 random*:ab,ti OR placebo:ab,ti OR 'double blind':ab,ti OR crossover:ab,ti OR 'cross over':ab,ti<br>#5 #1 AND #2 AND #3 AND #4	149 records
CENTRAL (Cochrane)	#1 NSAID* OR "non-steroidal anti-inflammatory" OR "nonsteroidal anti-inflammatory" OR aspirin OR indomethacin OR diclofenac OR naproxen OR ibuprofen OR celecoxib OR loxoprofen<br>#2 "small intestine" OR "small intestines" OR "small bowel" OR jejunum OR jejunal OR ileum OR ileal OR enteropathy OR enteropathies<br>#3 (mucosal NEXT injur*) OR (intestinal NEXT injur*) OR (intestinal NEXT damage) OR (intestinal NEXT permeab*) OR (capsule NEXT endoscop*) OR (video NEXT capsule) OR erosion OR erosions OR ulcer OR ulcers OR lactulose OR mannitol OR "Lewis score"<br>#4 #1 AND #2 AND #3	153 records (Trials only)
CNKI	Search A (Subject): (非甾体抗炎药 OR NSAIDs OR 阿司匹林 OR 吲哚美辛 OR 双氯芬酸) AND (小肠损伤 OR 小肠黏膜损伤 OR 小肠溃疡 OR 肠道通透性 OR 胶囊内镜)	40 records
Korean databases (KISS, OASIS, KMbase)	(NSAID OR aspirin OR indomethacin) AND (small intestinal injury OR intestinal permeability OR capsule endoscopy)	No eligible studies identified
Total	PubMed + Embase + CENTRAL + CNKI	438 records

Search conducted from database inception to January 2026, with no language restrictions. CENTRAL, Cochrane Central Register of Controlled Trials; CNKI, China National Knowledge Infrastructure; NSAID, nonsteroidal anti-inflammatory drug. English explanation of Chinese search terms: 非甾体抗炎药 = nonsteroidal anti-inflammatory drugs; 阿司匹林 = aspirin; 吲哚美辛 = indomethacin; 双氯芬酸 = diclofenac; 小肠损伤 = small intestinal injury; 小肠黏膜损伤 = small intestinal mucosal injury; 小肠溃疡 = small intestinal ulcer; 肠道通透性 = intestinal permeability; 胶囊内镜 = capsule endoscopy; KISS, Korean Studies Information Service System; KMbase, Korean Medical Database; OASIS, Oriental Medicine Advanced Searching Integrated System.
